# Arabinogalactan enhances *Mycobacterium marinum* virulence by suppressing host innate immune responses

**DOI:** 10.3389/fimmu.2022.879775

**Published:** 2022-08-26

**Authors:** Ye-yu Li, Han-Mei Liu, Decheng Wang, Yan Lu, Cairong Ding, Li-Shuang Zhou, Xiang-Yang Wu, Zi-Wei Zhou, Shu-qin Xu, Chen Lin, Lian-Hua Qin, Yao Li, Jun Liu, Hai-Peng Liu, Lu Zhang

**Affiliations:** ^1^ Department of Microbiology, School of Life Science, Fudan University, Shanghai, China; ^2^ State Key Laboratory of Genetic Engineering, School of Life Science, Fudan University, Shanghai, China; ^3^ School of Medicine, China Three Gorges University, Yichang, China; ^4^ Department of Natural Medicine, School of Pharmacy, Fudan University, Shanghai, China; ^5^ Shanghai Key Lab of Tuberculosis, Shanghai Pulmonary Hospital, Tongji University School of Medicine, Shanghai, China; ^6^ Department of Molecular Genetics, University of Toronto, Toronto, ON, Canada; ^7^ Shanghai Engineering Research Center of Industrial Microorganisms, Shanghai, China

**Keywords:** mycobacterial arabinogalactan, virulence, macrophages, innate immunity, CRISPRi

## Abstract

Arabinogalactan (AG) participates in forming the cell wall core of mycobacteria, a structure known as the mAGP complex. Few studies have reported the virulence of inartificial AG or its interaction with the host immune system. Using clustered regularly interspaced short palindromic repeats interference gene editing technology, conditional *Mycobacterium marinum* mutants were constructed with a low expression of *embA* or *glfT2* (EmbA_KD or GlfT2_KD), which are separately involved in the biosynthesis of AG arabinose and galactose domains. High-performance gel permeation chromatography and high-performance liquid chromatography assays confirmed that the EmbA_KD strain showed a remarkable decrease in AG content with fragmentary arabinose chains, and the GlfT2_KD strain displayed less reduction in content with cut-down galactose chains. Based on transmission and scanning electron microscopy observations, the cell walls of the two mutants were found to be dramatically thickened, and the boundaries of different layers were more distinct. Phenotypes including the over-secretion of extracellular substances and enhanced spreading motility with a concomitant decreased resistance to ethambutol appeared in the EmbA_KD strain. The EmbA_KD and GlfT2_KD strains displayed limited intracellular proliferation after infecting murine J774A.1 macrophages. The disease progression infected with the EmbA_KD or GlfT2_KD strain significantly slowed down in zebrafish/murine tail infection models as well. Through transcriptome profiling, macrophages infected by EmbA_KD/GlfT2_KD strains showed enhanced oxidative metabolism. The cell survival measured using the CCK8 assay of macrophages exposed to the EmbA_KD strain was upregulated and consistent with the pathway enrichment analysis of differentially expressed genes in terms of cell cycle/apoptosis. The overexpression of C/EBPβ and the increasing secretion of proinflammatory cytokines were validated in the macrophages infected by the EmbA_KD mutant. In conclusion, the AG of *Mycobacterium* appears to restrain the host innate immune responses to enhance intracellular proliferation by interfering with oxidative metabolism and causing macrophage death. The arabinose chains of AG influence the *Mycobacterium* virulence and pathogenicity to a greater extent.

## Introduction

Tuberculosis (TB) is a respiratory infectious disease caused by *Mycobacterium tuberculosis* (*M.tb*), and nearly a fourth of the global population is infected by *M.tb*. With the onset of the worldwide coronavirus disease 2019 (COVID-19) pandemic, the TB mortality rate appeared to increase in 2020 (1). A total of 1.5 million people (including acquired immune deficiency syndrome patients) died from TB, an increase of approximately 92,000 compared to that in 2019 (1–3). Many first- and second-line chemotherapeutic drugs are available (1, 4), yet the morbidity and mortality rates remain high due to the severe situation of multi-drug resistant tuberculosis and co-infection with human immunodeficiency virus (5–8). Given these circumstances, we need to pay intensive attention to the fundamental mechanisms of TB pathogenesis with the aim of optimizing anti-tuberculosis therapy.

A complex cell wall structure acts as a natural barrier between the slow-growing *Mycobacterium* and antibiotics and acts as the crucial virulence factor (2). Cross-linked peptidoglycan, highly branched arabinogalactan (AG), and characteristic long-chain mycolic acid form the mycobacterial cell wall core, known as the mAGP complex (2). Embedded with proteins, free lipids, and lipopolysaccharides, the hydrophobic cell wall skeleton is designed to prevent the entry of hydrophilic antibiotics and environmental stressors (3, 4). Cell walls can undergo dynamic transformation to adapt their structure to different environments and growth stages—for example, peptidoglycans could be structurally altered during division and resuscitation (5–7). Cell wall components can be identified as pathogen-associated molecular patterns (PAMPs) by host pathogen recognition receptors, such as Toll-like receptors (TLRs), C-type lectin receptors, and Nod-like receptors. These components can trigger host immune and inflammatory responses accompanied by the immune evasion of pathogens (8–10).

Interactions between the *Mycobacterium* cell wall and host cell are essential for immunomodulation (11, 12). Trehalose-6,6′-dimycolate, which is the most abundant cell wall glycolipid, binds to the C-type lectin MINCLE to activate the downstream PI3K-AKT-GSK3 and NF-κB signaling pathways and upregulate the levels of interleukin-6/12 (IL-6/12) and tumor necrosis factor alpha (13, 14). Mannose-capped lipoarabinomannan binds with the mannose receptor to facilitate the expression of IL-8, signal transducer and activator of transcription, and cyclooxygenase 2 and inhibit cell apoptosis, pro-inflammatory cytokine production, and phagocyte maturation (15–17). Peptidoglycan can be recognized by Toll-like receptors (TLR2/4) and NOD-like receptors (NOD1/2) (18–20). Meanwhile, *M.tb* evades immune recognition through the deacetylation of peptidoglycan (20). Owing to the dynamic variability and complexity of cell wall constitution and host microenvironment, the specific immunological effects of mycobacterial cell wall components remain to be further elucidated.

Unlike other cell wall components that have been extensively investigated, the virulent mechanisms of AG have been insufficiently investigated except for its detailed biosynthetic process. AG is composed of a galactose backbone and three highly branched side chains of arabinose, which are linked internally to peptidoglycans and externally to mycolic acid (4, 21). AG was found to show a strong potential for immune stimulation and regulation (22) and has been applied with Ag85B from *M.tb* as an immune adjuvant to strengthen lymphocyte proliferation and cytokine secretion (23, 24). On the other hand, AG isolated from *M.tb* was shown to inhibit the immune responses of lymphocytes in humans and guinea pigs (25–27). Chemically synthetic AG was proven to bind to the galactoside-binding protein galectin9, triggering the transforming-activated factor kinase 1/extracellular receptor kinase/matrix metalloproteins (MMPs) signaling axis and promoting the expression of MMPs, leading to aggravated lung damage and *M.tb* infection (28, 29).

The effects of AG on pathogenic mycobacteria virulence and underlying immune mechanisms, especially the roles of AG galactose backbone and arabinose branches, have not been clarified to date. Considering the clear AG biosynthesis pathway, it would be a feasible strategy to construct a series of mutants of AG biosynthesis-related genes, which correspond to different degrees of defects in AG structure. By comparing these mutants, the physiological functions of AG or its backbone and branch chains will be elucidated. *Mycobacterium marinum* is an aquatic pathogen and is highly homologous with *M.tb*, with over 3,000 homologous genes (30). The characteristic granuloma structure and development in zebrafish infected by *M. marinum* are highly similar to those in human TB (31). *M. marinum* presents 80% sequence identity with *M.tb* in terms of seven key genes (*aftA*, *aftC*, *aftD*, *embA*, *embB*, *glfT1*, and *glfT2*) involved in AG synthesis. High conservation in AG structure was also confirmed in both *M.tb* and *M. marinum* (32).

In this study, conditional knock-down *M. marinum* strains were constructed using clustered regularly interspaced short palindromic repeats interference (CRISPRi) with low expression levels of *embA* or *glfT2*. Changes in AG content/structure and cell wall structure transformation were observed in knock-down mutants. Through infection models *in vivo/in vitro*, AG was proven to be a virulence factor in the whole bacteria condition. The underlying immune mechanisms were also profiled using a macrophage infection model. Our findings confirm that AG and *embA* gene are important virulence determinants of *M. marinum* and recommend new targets for anti-tuberculosis immune intervention.

## Results

### *embA/glfT2* knock-down inhibited AG synthesis and caused the disruption of polysaccharide chains

**Figure 1:**
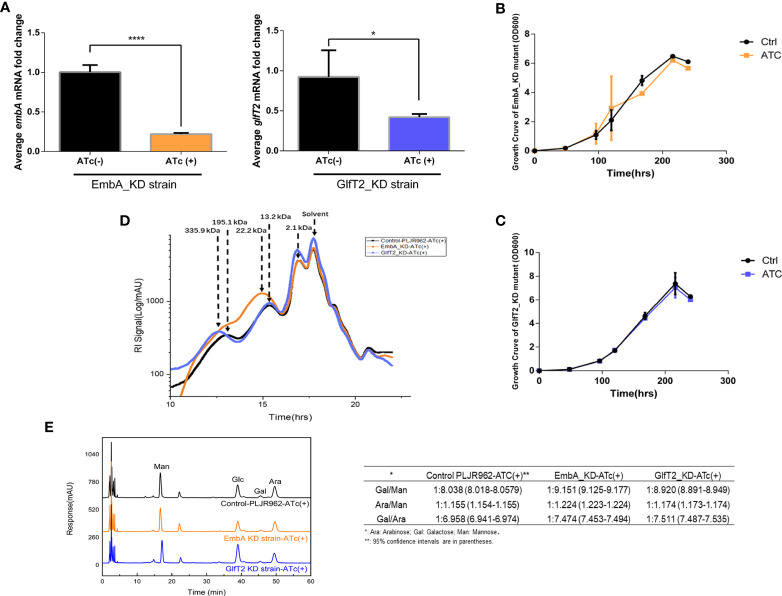
Construction of conditional EmbA/GlfT2_KD strains and validation of impaired arabinogalactan synthesis in *Mycobacterium marinum* cell wall. **(A)** qPCR proved the corresponding relative expression of *embA* or *glfT2* of *embA* knock-down (EmbA_KD) or *glfT2* knock-down (GlfT2_KD) strains with or without anhydrotetracycline (ATc) induction at logarithmic growth stage. Data are plotted as mean ± standard deviation (SD), unpaired *t*-test: ****p < 0.0001, *p = 0.0236. **(B, C)** Growth curves of EmbA/GlfT2_KD strain with or without ATc induction. Absorbance (OD_600_) measured in the bacterial liquid reflects the growth. **(D)** High-performance gel permeation chromatography was used to analyze the polysaccharide molecular weights of knock-down strain cell walls. The linear regression equation of retention time (*t*_R_) and relative molecular weight (MW) of polysaccharide is logMW = -0.5042 t_R_ + 11.884. Relative molecular weights corresponding to peaks are marked. **(E)** High-performance liquid chromatography was used to analyze the monosaccharide contents in the cell walls of control and knock-down strains. The peak area integral was used to calculate the proportion of each monosaccharide, and the ratios of arabinose–galactose/mannose or galactose/arabinose were calculated. The calculation results are presented in the table.

Most genes involved in AG biosynthesis are essential genes, and the gene knock-out was found to cause the stunted growth or death of *Mycobacterium* (33–37). CRISPRi technology with Cas9 from *Streptococcus thermophilus* (dCas9_Sth1_) achieves the robust knockdown of endogenous gene expression in *Mycobacterium* (38). Thus, in this study, genes with definitive functions in AG biosynthesis (*glfT1/glfT2/embA/embB/aftC*) were knocked down using CRISPRi. sgRNAs targeted to select genes were designed and inserted into the CRISPRi backbone (**Supplementary Figure S1** and **Supplementary Table S1**). A knock-down library under anhydrotetracycline (ATc) induction was thus constructed and validated by real-time reverse-transcriptase polymerase chain reaction (qRT-PCR) as shown in **Figure 1A** and **Supplementary Figure S2A**. Concurrently, the original plasmid without sgRNA coding sequence was electroporated into the *M. marinum* (M strain) to be used as the control strain. No significant influence from the original plasmid on gene expression and growth curve was found in the control strain (**Supplementary Figures S2B, C**). In agreement with the criterion of greater than a onefold downregulation of a stably expressed gene (unpaired *t*-test, *P*-value <0.05), *embA* and *glfT2* knockdown strains, whose target gene expression levels were reduced by approximately 70 and 55%, respectively (referred to as EmbA_KD and GlfT2_KD strain below), were carefully selected (**Figure 1A**). No significant differences in the growth curves of the two knock-down strains with or without the induction of ATc were noted (**Figures 1B, C**).

To explore the effects of *embA* and *glfT2* knock-down on AG content and/or structure, the molecular weights of polysaccharides in the cell wall were confirmed by high-performance gel permeation chromatography (HPGPC). The peaks appeared at 195.1 and 13.2 kDa in the control curves. While the curve of the EmbA_KD strain showed a peak at around 22.2 kDa, the peak shifted to 335.9 kDa in the GlfT2_KD strain (**Figure 1D**). Duplicable bacterial batches were detected to calculate relative ratios of monosaccharides by high-performance liquid chromatography (HPLC). Mannose was selected as the benchmark substance because it is independent of AG biosynthesis. The ratio of galactose to mannose in the EmbA_KD strain changed significantly from 1:8.038 [95% confidence interval (CI): 8.018–8.0579] to 1:9.151 (95% CI: 9.125–9.177), and that of the GlfT2_KD strain changed to 1:8.82 (95% CI: 8.891–8.949), while the arabinose/mannose ratio was 1:1.224 (95% CI: 1.223–1.224) in the EmbA_KD strain and 1:1.174 (95% CI: 1.173–1.174) in the GlfT2_KD strain compared with 1:1.155 (95% CI: 1.154–1.155) in the control (**Figure 1E**). On the other hand, the ratios of galactose to arabinose (Gal/Ara) were calculated to evaluate AG structural deficiency. GlfT2_KD strain had the highest Gal/Ara ratio of 1:7.511 (95% CI: 7.487–7.535), and EmbA_KD strain had a ratio of 1:7.474 (95% CI: 7.453–7.494) when compared with 1:6.958 (95% CI: 6.941–6.974) in the control (**Figure 1E**).

Three domains of D-arabinan, each composed of approximately 23 D-arabinofuranosyl, are affixed to the eighth, 10^th^, and 12th of ~30 C-5 hydroxyl of β(1→6)-linked galactofuranosyl AG backbone units (21). The arabinan domain also existed in lipoarabinomannan and occupied a larger proportion of cell wall monosaccharides than galactose (15). Ratio reduction of arabinose or galactose to mannose implied a deficient branched chain or main backbone, which caused shifted relative molecular weight peaks of polysaccharides in EmbA_KD/GlfT2_KD strains, as shown by the HPGPC assay results in **Figure 1**. A new question rising from the HPLC results should be answered: why arabinose and galactose reduced concurrently in two distinct knock-down mutants. The possibility of interaction or regulation between *embA* and *glfT2* genes was excluded since no relevance between their expression levels was found (**Supplementary Figures S2,E**). In the GlfT2_KD strain, it is probable that the low level of *glfT2* resulted in blocking galactofuranosyl main chain synthesis with the loss of the backbone for subsequent arabinofuranosyl branched chains, while in the EmbA_KD strain, undefined feedback regulatory mechanisms existed, in which blocked arabinofuranosyl chain synthesis delivers the signal that the galactose was no longer needed. Despite the speculative part, the maximum arabinose and galactose reductions from the HPLC results were still convincing in the EmbA_KD strain when compared with the GlfT2_KD strain.

Taking the HPGPC and HPLC results together, the *embA/glfT2* knock-down was inferred to inhibit the AG synthesis and break the AG chains, and a low expression of *embA* could lead to more severe effects.

### *embA*/*glfT2* knock-down led to cell wall thickening and other phenotypic differences

**Figure 2:**
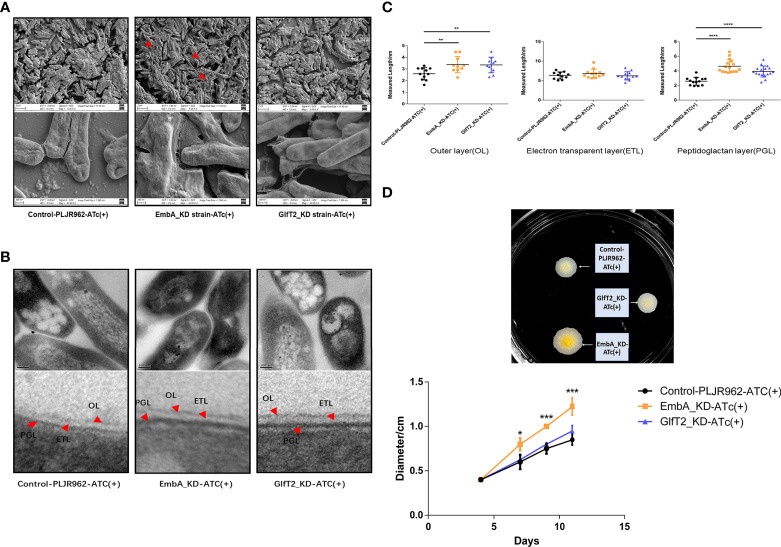
Cell wall structure of knock-down strains in the scanning electron microscopy (SEM) and transmission electron microscopy (TEM) fields. **(A)** The control PLJR962 strain and EmbA/GlfT2_KD strains were observed by SEM at ×10,000 and ×100,000 magnification. The red arrow indicates the extracellular material around the bacteria. **(B)** TEM observation of the control PLJR962 strain and EmbA/GlfT2_KD strains at ×100,000 magnification and partial enlargement of the cell wall. The red arrows indicate the outer layer (OL), electron transparent layer (ETL), and peptidoglycan layer (PGL). **(C)** A unified scale is used to measure the thickness of OL, ETL, and PGL layers of each strain from **(B)** in all visual fields by Image J software. The cell walls of three bacteria in each visual field were taken for measurement. Data are plotted as mean ± SD, unpaired *t*-test: **P < 0.01, ***P < 0.001, and ****P < 0.0001. **(D)** The diameters of plaques were measured on 7H9 agar plate at timepoints of 4/7/9/11 days to reflect the biofilm formation. The plaques on day 11 are shown in the upper figure. A total of three parallel plates were used for significance analysis using unpaired *t*-test: *P < 0.05 and ***P < 0.001.

To explore the novel morphological phenotypes arising from AG content reduction and structural defects, the cell wall of knock-down mutants was characterized by scanning electron and transmission electron microscopy (SEM and TEM, respectively). Secreted extracellular substance adhesion to EmbA_KD was observed under the SEM field, and the GlfT2_KD strain showed a smoother surface and less extracellular substances in contrast (**Figure 2A**). The cell walls of two knock-down mutants were observed to be dramatically thickened, and the boundaries of different layers were more distinct under TEM observation (**Figure 2B**). The image processing software Image J was used to measure the thickness of different cell wall layers with a uniform scale. After quantification, the outer layer thickness of the EmbA_KD and GlfT2_KD strains increased to 3.380 ± 0.2223 and 3.349 ± 0.2121 nm (compared with 2.595 ± 0.1569 nm of control), and the peptidoglycan layer thickened to 4.629 ± 0.2223 and 3.912 ± 0.1836 nm in the EmbA and GlfT2_KD strains, respectively, when compared with 2.583 ± 0.1488 nm in the control. It should be noted that the width of the electrophobic layer in which the AG was located did not significantly change (**Figure 2C**).

The surface spreading motility and biofilm formation of mycobacteria were affected by cell wall compounds or extracellular substances (39, 40). When bacterial droplets were added to the 7H9 agarose motility plate, the EmbA_KD strain formed colonies with larger diameters, which suggested greater migration capability (**Figure 2D**). Such a change in motility is not likely due to the enhanced proliferation among the strains, as no difference was found in the growth curves (**Figure 1B**). Secreted extracellular substances in the EmbA_KD strain could be the fundamental reason for the increase in sliding motility.

Mycobacterial resistance to some drugs also depends on cell wall structure (41). The drug resistance of knock-down strains to ethambutol (targeting *emb* gene) and isoniazid (targeting catalase independent to AG) were assessed using a minimum inhibitory concentration (MIC) assay. The MIC of EmbA_KD strain in terms of ethambutol was reduced to 5.24 μg/ml, while it was 20 μg/ml in the other two groups (**Supplementary Table S2**). Lower ethambutol doses blocked the mycobacteria cell wall biosynthesis in the EmbA_KD strain. Furthermore, the MIC of knock-down strains against isoniazid did not differ from the control.

In brief, in addition to AG content, the polysaccharide chain composition of AG was also found to affect the cell wall structure and other physiological characteristics of *M. marinum*.

### EmbA_KD and GlfT2_KD mutants are defective in intracellular replication

**Figure 3:**
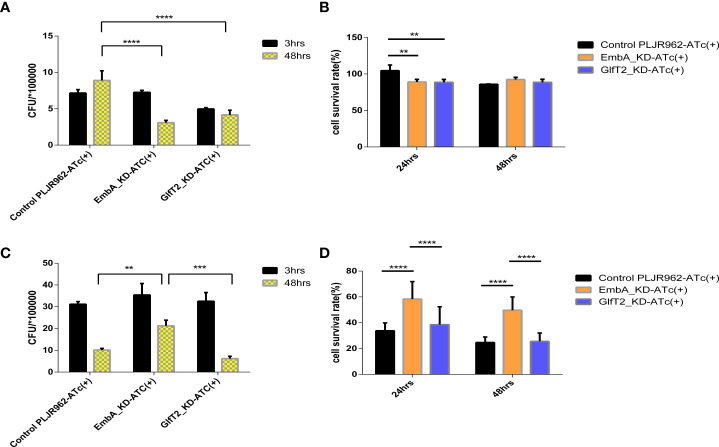
EmbA/GlfT2_KD strain-infected murine macrophage J774A.1 line. **(A, B)** Macrophages were infected with a multiplicity of infection (MOI) = 1 at 48-h timepoints, and cell survival rates were determined using the cell counting kit (CCK8) at 24/48 h. The cell survival rate was calculated as follows: survival rate = [(As - Ab)/(Ac - Ab)] × 100%, in which As is the absorbance of the infected well of the control of knock-down groups, Ab is the absorbance of medium-only well without added cells, and Ac is the absorbance of uninfected cell wells. **(C, D)** The macrophages were infected with a MOI = 5 at 48-h timepoints, and the cell survival rate was determined by CCK8 at 24/48 h. **(A–D)** Data processing is homogenized, and data are plotted as mean ± SD. Two-way ANOVA tests are used for all significance analysis here: **P < 0.01, **P < 0.01, ***P < 0.001, and ****P < 0.0001.

We chose a macrophage infection model to explore the potential impact of cell wall structure transformation induced by *embA* and *glfT2* knock-down on mycobacterial virulence. After murine J774A.1 macrophages were infected by *M. marinum* strains with multiplicity of infection (MOI) = 1, the intracellular growth of EmbA_KD, GlfT2_KD, and control strains were quantified by counting the colony-forming units (CFU). At 48 h post-infection, the control strain grew substantially to 8.904 ± 0.7610 × 10^5^ CFU. In contrast, growth inside macrophages of both EmbA_KD and GlfT2_KD was significantly reduced, reaching 3.089 ± 0.1847 × 10^5^ and 4.170 ± 0.3648 × 10^5^ CFU, respectively (**Figure 3A**). Meanwhile, the survival rates of macrophages were also assessed using the cell counting kit (CCK8) assay, and no difference was found among the infected and non-infected groups (**Figure 3B**). Considering the equivalent growth capabilities in 7H9 media exhibited by all three strains, the above-mentioned results indicated that the low expression of *embA* and *glfT2* could significantly disrupt the intracellular bacterial proliferation.

While macrophages infected with a higher MOI (= 5), the intracellular CFUs of the EmbA_KD strain were 1.71 ± 0.0289 × 10^6^ when compared with those of the control strain at 5.13 ± 0.578 × 10^5^ and the GlfT2_KD strain at 5.06 ± 0.418 × 10^5^ at 48 h post-infection (**Figure 3C**). The survival rate of macrophages infected by the EmbA_KD strain was 49.72 ± 3.406%, which was higher than the control (24.77 ± 1.416%) and the GlfT2_KD strain (25.65 ± 2.124%) at the 48-h timepoint (**Figure 3D**). It seems that intracellular proliferation of knock-down mutants changed with higher MOIs, a finding that indicated an inconsistent bacterial load. In contrast to an MOI = 1, a higher-MOI (=5) infection overwhelmed the macrophages and resulted in its necrosis or apoptosis, and the higher survival rate of macrophages infected by the EmbA_KD strain caused an increase in intracellular CFU, which reflected a decrease in the virulence of EmbA_KD. As for the GlfT2_KD strain, the decrease in the trend of the bacterial load in MOI = 1 also existed in MOI = 5, which was not reflected in the two-way analysis of variance (ANOVA) test but in the unpaired *t*-test (*p* = 0.0063), yet we preferred the two-way ANOVA test to engage the comparison of different timepoints.

Taken together, the virulence of the EmbA_KD strain declined remarkably in the macrophage infection model, and a relatively less reduction of virulence in the GlfT2_KD strain was detected.

### EmbA/GlfT2_KD mutants delayed disease progression in zebrafish and the EmbA_KD strain tended to be eliminated

**Figure 4:**
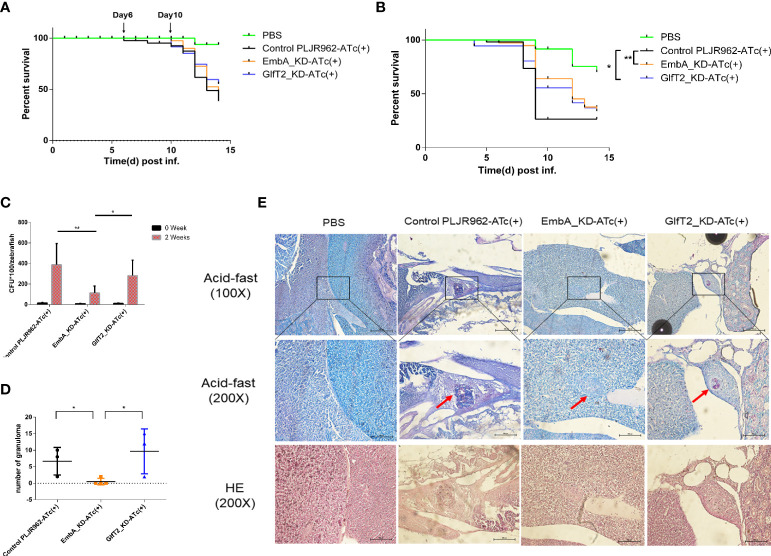
Zebrafish infection model with EmbA/GlfT2_KD strains. **(A, B)** Survival curves of larvae zebrafish infected by control PLJR962 strain and EmbA/GlfT2_KD strains with initial colony forming unit (CFU) = 100 or 300, respectively (*n* = 20–50). Log-rank tests are used to test the significance of the Kaplan–Meier survival curves: *P < 0.05, **P < 0.01. **(C, D)** CFU counts and the granuloma numbers in adult zebrafish infected by control strain and EmbA/GlfT2 knock-down strains, respectively, at 2 weeks post-injection. Data were plotted as mean ± SD. Two-way analysis of variance tests were used for all significance analysis: *P < 0.05 and **P < 0.01. **(E)** Acid-fast staining and hematoxylin and eosin (H&E) staining of zebrafish sections. Acid-fast staining was observed with an optical microscope at ×100 and ×200, and the visual field of H&E stain was the corresponding area of acid-fast staining at ×200 magnification. The red arrow points to the granuloma, and the purplish red color in the center of acid-fast granuloma represents the thallus.

Zebrafish (*Danio rerio*) is a natural host for *M. marinum* and has been widely used as a laboratory TB infection model. The larvae of zebrafish provide a powerful tool to investigate the innate immunity responses to *Mycobacterium* in the host (42–44). To assess the role of *embA and glfT2* in bacterial pathogenicity, larvae zebrafish (30–50 per group) were micro-injected with EmbA_KD, GlfT2_KD, or the control strain (100 CFU/fish) into the tails and were monitored for survival. The larvae death kick-offs were delayed to day 10 after the knock-down groups were infected, while the delay occurred on day 6 after the control strain infection (**Figure 4A**). When the injection dose was increased to 300 CFU/fish, the median survival time of fish infected with the control and GlfT2_KD strains was 8 days for both, whereas in fish infected with EmbA_KD, the time was 12 days. The log-rank analysis indicated that the survival curve of the larvae zebrafish group infected with the EmbA_KD or GlfT2_KD strain was significantly different from that of the control group (*p* < 0.01 or *p* < 0.05) (**Figure 4B**).

It is well known that the adaptive immune system of adult zebrafish offers a significant advancement in the establishment of an embryonic infection model (32), and the granulomas formed in adult zebrafish are very similar to those in human TB (45). We infected adult zebrafish intraperitoneally with 1,000 CFU/fish to observe bacterial proliferation and granulomatous formation *in vivo*. At 2 weeks post-infection, the bacterial burden in fish infected with the EmbA_KD strain was greatly reduced compared to those infected with control or the GlfT2_KD strain (**Figure 4C**). Fish infected with EmbA_KD had more than threefold lower bacteria load (11.2857 ± 2.5234 × 10^3^ CFU/fish) than those infected with control (38.7778 ± 6.8775 × 10^3^ CFU/fish) or the GlfT2_KD strain (28.1333 ± 3.9180 × 10^3^ CFU/fish).

Histopathology sections were read after acid-fast staining and hematoxylin and eosin (H&E) staining to analyze the pathological characteristics at 2 weeks post-infection (four fish per group were sacrificed). Consistent with the bacterial burden *in vivo*, zebrafish infected with control or the GlfT2_KD strain exhibited extensive bacterial dissemination in the livers and follicles. Significant amounts of organized granulomas were detected in these organs, and most of them presented a necrotic center; the bacteria were predominantly found inside granulomas with a few free bacilli detected (**Figures 4D,E**). By comparison, no discernible pathological damage in the livers or other organs of zebrafish infected with EmbA_KD was found. Only two aggregated granulomas were observed in the liver of the EmbA_KD group, while 20–29 granulomas were found in the other two groups (**Figure 4D**). The granulomas in the EmbA_KD group were characterized in the early stages by well-organized and less bacteria in the internal structures (**Figure 4E**).

From the zebrafish infection results, it is believed that the EmbA_KD strain was more easily eliminated by the host. The onset of progression of larvae zebrafish infected by the EmbA_KD and GlfT2_KD strains significantly slowed down. Two independent replicates were performed, and all results were reproducible. The time window was designed to be less than 2 weeks to minimize the effect of ATc invalid *in vivo* and to ensure the data to be as meaningful as possible.

### EmbA_KD/GlfT2_KD mutant strain infection attenuated lesions in murine tails

**Figure 5:**
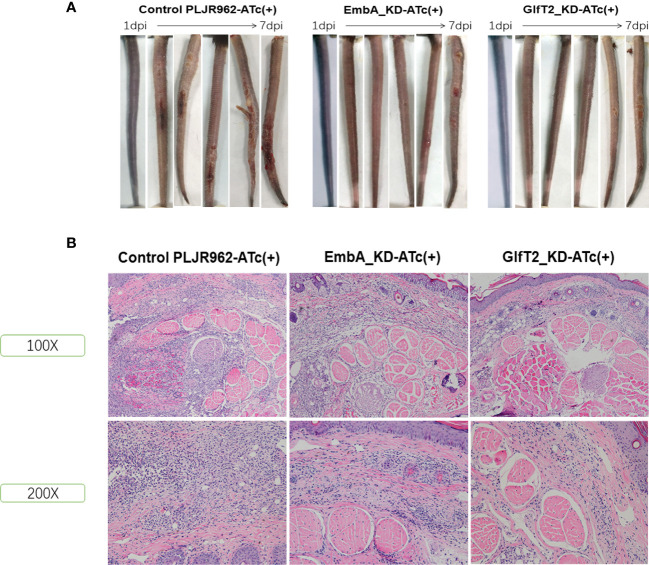
Murine tail infection model with EmbA/GlfT2_KD strains. **(A)** Tail ulcer formation in mice injected with control PLJR962 strain and EmbA/GlfT2_KD strains within 1 week post-injection. **(B)** Pathological H&E staining in mice ulcer. With distinguishable tail tissue cells, most of the clustered purple nuclei belong to lymphocytes.

Mice are the most frequently used animal model for tuberculosis fundamental research and drug development (46). Early studies demonstrated that *M. marinum* could infect cooler anatomical regions in mice, such as the tail, as 30 to 32α is the optimal temperature for *M. marinum* growth and beneficial to infective focus formation (47, 48). To validate the pathology of the EmbA_KD and GlfT2_KD mutant strains, mice were infected with *M. marinum* strains *via* tail vein injection, and disease progression was observed continuously for 1 week (*n* = 5 per group; the most ulcerated tail in each group was captured) as shown in **Figure 5A**. Visible tail lesions appeared at 2 days post-infection, with eventual formation of severe ulcerations, in the control strain. While the mice infected with the EmbA_KD or GlfT2_KD strains developed minor lesions with a concomitant delayed onset at 5 days post-infection. The degrees of inflammation of the murine tails were evaluated and distinguished qualitatively by histological H&E staining (**Figure 5B** and **Table 1**). The pathological analysis showed that the quantities of lymphocytes in these lesions were reduced, and moderate to little localized/focal histological features of inflammation were found in the knock-down groups. Severe and multifocal inflammations marked by the presence of lymphocytes and macrophages were observed in tails infected with the control strain, in addition to extensive tissue damage and vascular involvement (**Table 1**).

**Table 1:** Histopathology evaluation of infected mice’ tails.

	Degree of inflammation	Tissue damage	Vascular involvement	Inflammatory cell type
Control-PLJR962-ATc(+)	+++	+++	++	NEU, MΦ, LYM, and PC
EmbA_KD-ATc(+)	++	++	+	NEU, MΦ, LYM, and PC
GlfT2_KD –ATc(+)	+	+	+	NEU, MΦ, LYM, and PC

+, degree of severity; LYM, lymphocyte; MΦ, macrophage; NEU, neutrophil; PC, plasma cell.

Consistent with the defective replication in macrophages and delayed disease progression in larvae zebrafish, reduced pathological damage in murine tail after infection of the EmbA/GlfT2_KD strain further proved that *embA* and *glfT2* genes are the determinants of bacterial virulence. After integration with the catalytic function of *embA* and *glfT2* in AG biosynthesis, it is suggested that the arabinose chain and galactose chain of AG are key virulent factors in *M. marinum*.

### Oxidative metabolism and cell cycle were distrupted after EmbA_KD mutant infection based on transcriptome profiling

To gain insight into the underlying mechanism involved in the above-mentioned process, we utilized RNA-seq technology to profile the differentially expressed genes (DEGs) of murine J774A.1 macrophages infected with the EmbA_KD, GlfT2_KD, or control strain at 12 h post-infection. RNA sequencing data have been uploaded to the Gene Expression Omnibus database (GES197488).

**Figure 6:**
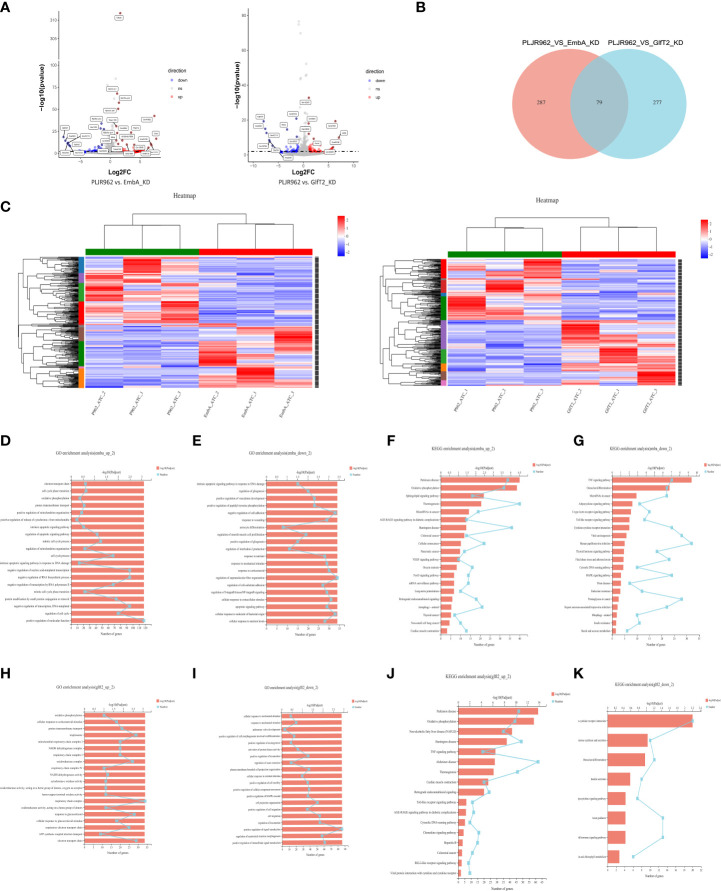
Transcriptome analysis of EmbA/GlfT2_KD strain-infected murine macrophage line J774A.1 (MOI = 5, 12 h). **(A)** Volcanic map of the differential quality of differentially expressed genes (DEGs). The abscissa is the multiple of the gene/transcript expression difference between the two samples, and the ordinate is the statistical test value of gene expression difference. A larger -log10 (*p*-value) indicates a more significant difference in expression. The values of horizontal and vertical coordinates were logarithmically processed. Each dot represents a specific gene. The software edgeR was used for DEG analysis, and the screening threshold was |log2FC| ≥ 1 and the *p*-value was <0.05. **(B, C)** Venn diagram and clustering heat map of DEGs in the two groups of knock-down strains compared with the control group, and there are 10 subclusters in both groups. Each column represents a sample, and each row represents a gene. The color in the figure denotes the expression level of the gene after a standardized treatment in each sample; red indicates a high expression level, while blue means a low expression level. On the left side of the heat map is the tree diagram of gene sub-clustering, and on the right side is the name of the genes. The upper part represents the tree of sample clustering. The closer the branches of the two samples are, the closer the variation trends of the gene expression levels are. **(D, E)** Gene ontology (GO) enrichment analysis of genes up/downregulated in macrophages infected with the EmbA_KD strain. The ordinate represents the GO term, and the lower abscissa represents the number of genes/transcripts compared to the GO term, corresponding to different points on the broken line. The upper horizontal coordinate represents the significance level of enrichment, corresponding to the height of the column. A smaller *p*-value (adjusted) indicates a larger value of −log10 (*p*-adjusted); thus, the more significant it is means that the GO term is enriched. **(F, G)** Kyoto Encyclopedia of Genes and Genomes (KEGG) pathway enrichment analysis of genes up/downregulated in macrophages infected with EmbA_KD strain. The vertical axis represents the KEGG pathway, and the horizontal axis represents the number of genes/transcripts compared to this pathway, corresponding to the different points on the broken line. The upper horizontal coordinate represents the significance level of enrichment, which corresponds to the height of the column. A smaller false discovery rate means a larger −log10 (*p*-adjusted) value and a more significantly enriched KEGG pathway. **(H, I)** GO enrichment analysis of genes up/downregulated in macrophages infected with the GlfT2_KD strain. **(J, K)** KEGG pathway enrichment analysis of genes up/downregulated in macrophages infected with the GlfT2_KD strain.

A total of 71.78 GB cleaned data from nine samples (three biological replicates per group) was obtained. DEGs were analyzed by *edgR*, and the cutoff threshold was analyzed using |log_2_FC| ≥1 and a *p*-value <0.05 (**Figure 6A**). A total of 366 DEGs were detected between macrophages infected by the EmbA_KD and control strains, including 173 upregulated and 193 downregulated genes (**Supplementary Tables S4, S5**), and 356 DEGs between macrophages infected by the GlfT2_KD and control strains, including 185 upregulated and 171 downregulated genes, were found (**Supplementary Tables S6, S7**). Seventy-nine DEGs were found to have overlapped in two groups (**Figure 6B**). The overexpressed gene *zxda* and the downregulated gene *ugt1a8* stood out from the DEGs with the biggest fold changes in both knock-down mutant groups (**Figure 6A**). In addition, genes such as *pagr1a/tmem160/cebpb* exhibited an upward trend in the macrophages infected with the EmbA_KD strain, and genes, *e*.*g*., *ugt1a2*, showed the opposite trend.

DEG clustering analysis was performed among parallel control or mutant samples, and 10 sub-clusters each were stratified in the EmbA_KD or GlfT2_KD infection group (**Figure 6C**). Between the control and EmbA_KD infection groups, gene ontology (GO) analysis of upregulated DEGs indicated gene enrichment correlating to biological processes (BP) or molecular functions (MF), including the regulation of cell cycle, cell cycle process, mitotic cell cycle process, regulation of apoptotic signaling pathway, electron transport chain, and oxidative phosphorylation (**Figure 6D**). Meanwhile, the downregulated DEGs were enriched in BP/MF, namely, regulation of supramolecular fiber organization, negative regulation of cell adhesion, and cellular response to the molecule of bacterial origin (**Figure 6E**). Kyoto Encyclopedia of Genes and Genomes (KEGG) pathway enrichment analysis was also carried out to reveal the over-expressed pathways in the EmbA_KD infection group, namely, oxidative phosphorylation, vascular endothelial growth factor, signaling pathway, and FoxO signaling pathway and downregulated pathways, such as TNF/C-type lectin/Toll-like receptor/mitogen-activated kinase signaling pathways (**Figures 6F, G**).

The same analysis was performed for DEGs in the GlfT2_KD group *versus* the control group. The GO enrichment analysis suggested that a series of cellular physiological functions in terms of respiratory chain complex and oxidoreductase activity was enhanced in macrophages infected with the GlfT2_KD strain, while genes related to cell migration and signal transduction displayed a downward trend (**Figures 6H, I**). Besides this, the TNF/Toll-like receptor/chemokine/retinoic acid-inducible gene-like receptor signaling pathway, which is associated with an increase in inflammatory responses, increased in the GlfT2_KD group, and downregulation of the cytokine–cytokine receptor interaction pathway had occurred (**Figures 6J, K**).

Dampened oxidative phosphorylation and reprogrammed mitochondrial metabolism can be triggered in macrophages by mycobacteria to limit intracellular redox level and favor infection (49–52). On the basis of RNAseq analysis, elevated oxidative metabolism in macrophages could be correlated with impaired intracellular proliferation of knock-down mutants. Furthermore, the upregulated DEGs in EmbA_KD-infected macrophages were also enriched in cell cycle regulation, consistent with the result of the CCK8 assay (**Figure 3D**).

According to these findings, a hypothesis was proposed in which AG, especially arabinose domain, may play a role in promoting *M. marinum* intracellular survival by substantial intervention in oxidative metabolism and/or macrophage cell cycles.

### Expression of C/EBPβ and secretion of inflammatory cytokines were upregulated in macrophages infected with the EmbA_KD mutant

**Figure 7:**
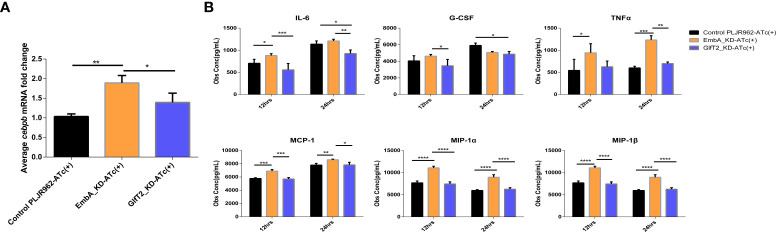
Validation of *cebpb* expression and secreted regulatory cytokine levels in an infected macrophage. **(A)** Real-time polymerase chain reaction was used to verify the mRNA expression level of *cepbp* in the corresponding sample of transcriptomes (from macrophages infected with multiplicity of infection = 5 and 12 h). Data were plotted as mean ± SD, unpaired *t*-test: *p = 0.0465 and **P = 0.0018. **(B)** Cytokine contents in the cell supernatant of macrophage J774A.1 infected by control PLJR962 strain and EmbA_KD strain with MOI = 5. Two-way ANOVA tests are used for all significance analysis here: **P < 0.01, **P < 0.01, ***P < 0.001, and ****P < 0.0001.

No clear explanation for the immune regulation of knock-down strains could be concluded from the enrichment analysis, and more evidence from further validation is needed. When we comprehensively considered DEGs on the basis of statistical differences and molecular function, the *cebpb* gene attracted our interest as it was significantly upregulated in macrophages infected with the EmbA_KD strain with a minimum *p*-value in the DEG list (**Figure 6A**). In this study, the expression levels of the *cebpb* gene were validated by qRT-PCR, and it showed a twofold upregulation (**Figure 7A**).

CCAAT/enhancer binding protein beta (C/EBPβ) encoded by *cebpb* is a transcription factor that is highly expressed in myelomonocytic cells and macrophages, is involved in the transcriptional regulation of cytokines (such as TNF/IL-6/MIP-1α/MIP-1β), and has anti-bacterial effects (53–55). Bacterial or other microbial products can activate or inhibit C/EBPβ *via* distinct signal transduction pathways (55). To reveal the cytokine expression profiles which are associated with C/EBPβ putatively, we detected 23 cytokines secreted by infected macrophages using a liquid suspension protein microarray.

Considering the time window of cytokine synthesis and secretion, 12 and 24 h after infection were chosen to detect the cytokine secretion levels. Four cytokines—namely, MCP-1, MIP-1 α, MIP-1β, and TNF-α—were upregulated in macrophages infected specifically by the EmbA_KD strain at both timepoints, and the IL-3, -6, -13, and -17 and granulocyte-macrophage colony-stimulating factor levels were upregulated only at 12 h post-infection (two-way ANOVA test, *p*-value <0.05) (**Figure 7B** and **Supplementary Figure S3**). In contrast, IL-6 and granulocyte-colony stimulating factor (G-CSF) were decreased significantly at 12 and 24 h post-infection with GlfT2_KD.

Upon combining the *in vivo/in vitro* infection results, it appears that the arabinose deficiency of EmbA_KD could promote the C/EBPβ expression in macrophages and enhance the inflammatory responses to inhibit the intracellular proliferation of *M. marinum*. Increased chemokine levels also helped recruit a variety of immune cells and improved the anti-mycobacterial ability *in vivo*. Eventually, as confirmed in zebrafish and mice infection models, the pathogenicity of EmbA_KD was found to be reduced.

## Discussion

The pathological progression of TB is initiated with a respiratory infection, after which it progresses to granulomas, tissue fibrosis, and cavities in the lung (56, 57). In the chronic infection process, the host immune system can recognize and kill *Mycobacterium*. On the other hand, bacteria will deceive or train immune cells to elude sterilization. Identifying key bacterial virulence regulators and investigating the underlying immune mechanisms will allow for a more thorough explanation of TB pathogenesis.

Our study showed that *embA* and *glfT2* play a critical role in the disease progression of *M. marinum* infection. First, the EmbA_KD and GlfT2_KD mutants led to a reduction in mortality in larvae zebrafish as demonstrated by the longer median survival time and better survival outcomes during an infection. It is suggested that a low expression of *embA* or *glfT2* in *M. marinum* caused the mutants to be more susceptible to early clearance by host innate immunity. Second, granulomas induced by EmbA_KD infection were much fewer and less well-organized with less bacilli inside, which prevented bacterial dissemination in adult zebrafish. The attenuation phenotype suggested that the bacterial multiplication of mutant *M. marinum* with a low expression of *embA* could be controlled successfully by adult zebrafish. Third, in the mice tail infection model, the EmbA_KD or GlfT2_KD infections developed very few and smaller lesions with a concurrent delay in onset. Inflammation in the lesion was reduced, and the inflammatory infiltrate in the dermis was attenuated in infected mice. It was further confirmed that the regulatory effects of *embA* and *glfT2* were relevant in the pathogenesis of *M. marinum*. Moreover, due to the co-transcription of the *embA* and *embB* genes (35), the expression of *embB* decreased along with the knock-down of *embA* in the EmbA_KD strain (**Supplementary Figures S2D,E**). The EmbA–EmbB–AcpM2 complex is the target of ethambutol, and the over-expression of *embA* and *embB* is associated with a high-level ethambutol resistance (41, 58–60), so it was reasonable to find a reduction in the resistance of the EmbA_KD strain to ethambutol in this study. The co-transcription of *embA* and *embB* could also explain why the decline of *embA* had a greater impact on *M. marinum* virulence than *glfT2*. Taken together, our study provides fundamental evidence concerning bacterial virulence factors and their pathogenic mechanisms, which is necessary for the development of novel interventions targeting TB.

More clues could be provided from the comparison of cell wall biochemical characteristics between the two knock-downs strains. The AG content and structure differences in the two knock-down strains were found as follows: (1) the EmbA_KD strain had a higher proportion of reduction in AG content and severely impaired arabinose chains and (2) the GlfT2_KD strain showed a lower degree in AG reduction with damage to the main chain of galactose. A significant loss of AG, especially arabinose, caused an extracellular leakage in the EmbA_KD strain, while the damaged galactose chain from depressed *glfT2* expression created a smooth cell wall surface. Defective AG synthesis in *embA* and *glfT2* knock-down strains led to a thickened mycobacterial cell wall structure. It is speculated that the connection between layers of cell wall became loose. The inner and outer layers lost structural attachment points from AG, which led to a thickened cell wall. The impact of extracellular leakage and/or unconfirmed cell wall structure alterations needs to be addressed and further explored as well.

On the other hand, the underlying immune mechanism was explored. In the host immune system, pioneer macrophages phagocytize *Mycobacterium* and trigger an antimicrobial response by producing pro-inflammatory cytokines such as TNF-α, IL-6, and reactive oxygen species (49), after which the process of killing bacilli is initiated (61). When comparing the transcriptome data in this study with those of another study, in which macrophages were exposed to a pure AG compound (28), unbiased DEG enrichment analysis highlighted the overlapping pathways in cell growth and death, oxidative phosphorylation, and signal transduction. Although more subsequent validations are needed, we assume that the elevated oxidative metabolism in macrophages infected by knock-down mutants contributes to the impaired intracellular proliferation of *M. marinum*. In parallel, improved cell viability and restricted apoptosis after infection by mutants caused a noticeable suppression of mycobacterial survival in phagocytes (62). An altered macrophage cell cycle also contributed to the reduced virulence and quick elimination of the EmbA_KD strain *in vivo/vitro* (63) as proven by CCK8 assay and CFU counting results. Though chemically synthetic AG was proven to bind to galectin9 (28), the recognition mechanism of native AG in the *Mycobacterium* is not yet fully understood, not to mention when narrowing down the galactose backbone and arabinose branches in AG separately. Due to finding that the cell wall was recognized as a whole, demystifying AG effects on the virulence in living bacteria could help to reveal the pathogenic mechanisms of AG in real life.

The TLR 2/4 signaling pathway can recognize PAMPs and regulate macrophage C/EBPβ expression during *M.tb* or *M. marinum* infection (64). To explore the recognition capacity of TLR2/4 for AG, we pretreated macrophages with TLR2 and TLR4 inhibitors and then measured the uptake and intracellular proliferation of the EmbA_KD mutant. Both TLR2 and TLR4 inhibitors had no influence on the uptake of the control strain, while the uptake of the EmbA_KD strain was inhibited by the TLR2 inhibitor, C29, but not by the TLR4 inhibitor, TAK242 (**Supplementary Figure S4**). It is noteworthy that the quantity of intracellular viable bacteria of the control strain significantly increased along with the inhibition of the TLR4 signaling pathway, yet the EmbA_KD strain did not change. These results imply that the entrance process into macrophages by the EmbA_KD strain was mediated by the TLR2 signaling pathway, and *embA* gene knock-down could assist *M. marinum* to elude TLR4-dependent bacterial killing. Castro-Alves *et al.* reported that liner arabino-oligosaccharides with a higher degree of polymerization could enhance the release of TNF-α/IL-6/MCP-1 through the TLR4/Myd88 pathway (65). The EmbA_KD strain with a lower degree of polymerization in arabinose may omit the biased activation of the TLR4 pathway.

We also speculated that upregulated *cebpb* level facilitates pro-inflammatory cytokine (such as IL-6, MCP-1, MIP-1, and TNF-α) secretion in macrophages. A fortified colony and chemotaxis of other macrophages or monocytes would be induced by cytokines and chemokines, such as MCP-1 and MIP-1, which was critical to recruit macrophages in mycobacterial infection (66–68). Adaptive immunity was then initiated, and granulomas began to form following the recruitment of macrophages and monocytes (69). Consistent with the histology results observed in adult zebrafish infected with the EmbA_KD strain, additional macrophages and monocytes were recruited by chemokine over-secretion, after which they participated in the formation of well-organized granulomas and limited bacterial spread in adult zebrafish. Other pro-inflammatory cytokines such as TNF-α/IL-6 also engaged in limiting *Mycobacterium* infection (45, 70).

As for the interpretation in the attenuated virulence of the EmbA/GlfT2_KD strain, which was concluded from suppressed replication *in vitro* (MOI = 1) and delayed onset or weakened inflammation in larvae zebrafish/murine tails, more clues will be provided by DEGs or secreted cytokines of macrophages—for instance, the zinc finger X-linked duplicated A (ZXDA) exhibited the maximum upregulated mRNA level in macrophages infected with two knock-down mutants. ZXDA is involved in forming a complex binding class II major histocompatibility complex trans activator and regulates MHC II gene transcription, with results that have been confirmed to be associated with latent *M.tb* infection and macrophage polarization (71–73). G-CSF is a candidate biomarker positively correlated with active tuberculosis (74), so the decreased secretion of G-CSF in macrophages infected by the EmbA_KD/GlfT2_KD strains suggests that AG may be associated with active tuberculosis.

Ultimately, an aggressive hypothesis was proposed when we refocused on the same and different changes in AG content and structure in the two knock-down mutants. AG acts as a virulence factor in restraining host innate immune response through reprogramming the oxidative metabolism of macrophages. Therein, arabinose chains contribute more to AG virulence. The polysaccharide domain directs the macrophage toward a specific fate and is ignored by the TLR2-C/EBPβ–inflammatory cytokine axis, as a broken-branched arabinose chain yields easier recognition and removal of *M. marinum*.

Slow disease progression in AG-related knock-down mutants demonstrates relevant clinical meanings. The primary adverse effects of ethambutol include optic neuritis and central nervous system toxicity (75). The reduction of ethambutol resistance in the EmbA_KD strain may suggest a novel solution in reducing drug resistance and dose when considering *embA* as the target of a combination or supplement strategy. Knocking down *embA/glfT2* can be also used for the construction of other attenuated strains, such as recombinant BCG with higher safety or *M.tb* with lower virulence. Each coin has two sides; the potential cytokine storm or septic shock should be of concern as well when considering the elevated inflammatory response triggered by the recombinant BCG strain. Inhibitors targeting GlfT2 have currently been widely evaluated as the novel therapy (76). Delayed disease progression, caused by the GlfT2_KD mutant in this study, confirmed that the target GlfT2 is valuable to translate to clinical medicine.

## Materials and methods

### Bacteria

*M. marinum* (laboratory *M* stains, resuscitated form a frozen stock) as the source of knock-down strains was grown in Middlebrook 7H9 broth (DifcoTM) with 10% acid–albumin–dextrose–catalase (ADC) (Becton Dickinson) or on 7H10 agar supplemented with 0.5% glycerol and 10% oleic acid–albumin–dextrose–catalase (Becton Dickinson) at 30α under aerobic conditions. Tween-80 was not added to the liquid culture because of its disruptive effects on the cell wall.

### Mutant construction by CRPSPRi

CRISPRi backbone PLJR962 was amplified in *Escherichia coli* (50 μg ml^-1^ kanamycin selection), digested with BsmBI (NEB, 55α for 4 h), and gel-purified prior to cloning the sgRNAs. Based on the PAM sequence (5′-NNAGAAW-3′) in the promoter/5′-UTR/open reading frame of seven target genes (*aftA/aftC/aftD/embA/embB/embB/glfT2*), which were involved in *M. marinum* AG biosynthesis, 20-nt bases of the template chain after PAM sequences were selected as the sequence encoding sgRNA oligos. The bases (GGGAG) were added to the 5′ end of the sgRNA coding sequence as the forward primer. Reverse primers came from a reverse complementary sequence with additional bases (AAAC) at the 5′ end and a single base C at the 3′ end. All primers were synthesized by Tsingke Biotechnology. Viscous ends formed after annealing and were connected *via* enzyme digestion of the skeleton. Forward and reverse primer sequences of the sgRNA-coding DNA were designed and are shown in **Supplementary Table S1**.

The annealing systems of sgRNA oligos, ligation process, and multi-gene knock-down plasmid construction were all modeled on the provided method (38). Successful insertion plasmids were sequenced by the primer 1834 (5′-TTCCTGTGAAGAGCCATTGATAATG-3′) and electroporated into the competent *M. marinum*. dCas9 protein expression was induced by ATc (100 ng/ml anhydrotetracycline) to prevent the transcription of target genes. When the bacteria grew to the middle logarithmic growth stage (optical density, OD_600_ = 2.0), RNA was extracted and was measured by fluorescence quantitative PCR. The primers are shown in **Supplementary Table S3**.

### HPGPC assay

Bacterial suspension (5 L, OD_600_ 2.0) was centrifuged and precipitated, after which the supernatant was removed. Phosphate-buffered saline (PBS, 200 ml) was added to resuspend the suspension in a water bath (80α for 30 min). The precipitates were centrifuged, washed again with PBS, and then lyophilized. The samples were dissolved in water (solid/liquid = 1:50) and boiled twice for 2 h each time. After centrifugation and chilling, the supernatants were obtained. Complex enzyme (2%) was added and reacted (55α for 2 h). After the reaction, the samples were boiled for 10 min to deactivate the enzyme. Supernatants were obtained again after centrifugation, precipitated with anhydrous ethanol (4×), and chilled (4α for 48 h). The precipitated component after centrifugation was dissolved in water and lyophilized again to obtain polysaccharides from the mycobacterial cell wall.

The relative molecular weights of the polysaccharides were determined by HPGPC using HPLC 1200 system. Polysaccharide samples (4 mg) were taken from the three groups, dissolved in ammonium acetate (20 mM, 1 ml), and then filtered through 0.22-μm membranes. Separation was achieved on TSK GMPWXL columns. Ammonium acetate aqueous solution (20 mM) was used as mobile phase at a flow rate of 0.6 ml/min. An aliquot of 20 μl solution was injected for analysis.

### Pre-column PMP-HPLC assay

Monosaccharides derived by PMP were identified by HPLC. Trifluoroacetic acid (2 M, 4 ml) was added to the polysaccharide samples (4 mg for each sample) and hydrolyzed in an oven (110α for 4 h). Methanol was added to the chilled samples several times to remove trifluoroacetic acid. The residues were then dissolved in deionized water (200 μl).

Hydrolyzed sample and standard solution were added to the centrifuge tube, respectively. Then, sodium hydroxide solution (100 μl, 0.6 M) and PMP methanol solution (200 μl, 0.5 M) were mixed and swirled with the samples. The cocktail was reacted in a water bath (70α for 100 min). Hydrochloric acid solution (200 μl, 0.3 M) was added for neutralization, and deionized water (400 μl) and chloroform (1 ml) were added for extraction. The extraction procedures were repeated twice after centrifugation. Microporous membranes (0.22 μm) were filtered and loaded into a liquid flask for detection by HPLC detection. The mobile phase consisted of 83% liquid A (0.02 M, phosphate buffer: KH_2_PO_4_, pH 6.86) and 17% liquid B (acetonitrile) at a flow rate of 1 ml/min. Separation was achieved on the chromatographic column C18, and the DAD detector was at a wavelength of 254 nm (30α).

### Morphology

Knock-down strains were inoculated into 7H9 medium (10% ADC; kanamycin, 25 μg ml^-1^), induced by ATc (200 ng ml^-1^), and then cultured at 30α for medium logarithmic growth (OD_600_ = 2.0). An equal amount of phosphate buffer (PB, 0.1 M, pH 6.5–7.0, including sodium dihydrogen phosphate and disodium hydrogen phosphate) was added to the bacterial broth. The PB buffer contained 1% heparin sodium (12,500 U/2 ml) and was mixed for 10 to 20 s. After centrifugation (2,000 rpm for 5 min), the supernatant was discarded. PB buffer was added, and the bacteria were shaken and mixed until no clumps were observed. A fixation solution (2% paraformaldehyde + 2% glutaraldehyde) was added to the samples, which were then centrifuged (4α at 2,000 rpm for 10 min). It should be noted that the centrifugation process of the samples for TEM observation was done at room temperature. The samples were centrifuged again and placed at 4α for 4 h. SEM (Zeiss GeminiSEM 300) and TEM (JEOL JEM-1230) were used for observations.

### Minimal inhibitory concentration assay

Strains were cultured in 7H9 medium (kanamycin, 25 μg ml^-1^, until OD_600_ = 0.6). ATc (200 ng ml^-1^) was added to induce knock-down for 2 to 3 days, and OD_600_ was monitored. The culture medium (OD_600_ = 0.5) was diluted (1,000×) in the 96-well plate. The drug concentration was twice the gradient dilution (the ethambutol concentration gradient was changed to 0.8 times gradient after the pre-experiment). The ATc concentration in the liquid environment remained constant. The strains and two drugs (ethambutol and isoniazid) in each group were repeated twice, and the drug-free wells were left as the control wells. After the planking, they were incubated at 30α for more than a week.

If bacterial precipitation at the bottom of the well was noted, the result was considered positive, and it is considered negative if clear. The drug concentration corresponding to the initial negative well was considered the minimum inhibitory concentration.

### Motility

Single bacterial suspensions were diluted with 7H9 medium (OD_600_ = 0.5), added (0.5 μl) to the surface of a motility medium (Middlebrook 7H9 broth solidified with 0.3% agarose), and then left at room temperature for 1 h until the bacterial liquid was blown dry. The plates were sealed with parafilm and incubated at 30α. The spreading capability of mycobacteria on the plate was observed by measuring the diameter of the drip plaque from the fourth day. The ATc concentration was kept unchanged during the whole process, and three parallel settings were monitored.

### Macrophage infection

Murine macrophages (J774A.1 cell line) were infected by knock-down strains with MOI = 1 or 5. The cells were washed with Dulbecco’s modified Eagle’s medium (DMEM, Gibco) and 10% fetal bovine serum three times to remove extracellular bacteria at 3 h post-infection. The remaining extracellular bacteria were killed using penicillin (100 U/ml) and streptomycin (100 μg/ml) in the medium. The ATc concentration in the culture medium was maintained at 200 ng/ml during or after infection to ensure the continued low expression of EmbA/GlfT2_KD stains. The cells were then incubated at 30α with 5% CO_2_ until further analysis.

The infected macrophages were lysed with 0.1% Triton X -100 (Sigma-Aldrich) for 10 min at room temperature. Tenfold serial dilutions of lysates were plated on 7H10 agar plates for CFU counting. A cell counting kit (CCK8, Yeasen) was used to calculate the survival rates. The cells (10,000 cells/100 μl per well) were spread on a 96-well plate. At the same time, uninfected cells and control wells without added cells were set up. CCK8 solution (10 μl) was added to each well, and the absorbance at 450 nm was measured with a microplate analyzer.

In the TLR signaling pathway blocking experiments, the cells were pre-treated with C29 (150 μM, MedChemExpress) and TAK242 (100 nM, Apexbio) for 1 h. The concentrations of C29 and TAK242 remained constant in the working fluid and DMEM medium.

### Larvae and adult zebrafish infection

Zebrafish embryos (*AB/TU*) were hatched into larvae and were anesthetized with tricaine methanesulfonate (500 ng/ml) 2 days after incubation. A single bacterial broth was diluted with PBS and added with phenol red. The bacterial liquid was then injected into the caudal vein through microinjection, and droplets were taken to dilute for CFU counting at the same time. Each fish was injected with 300 CFU of mycobacteria, and each group of strains was injected with 30 to 50 juvenile fish. After the injection, the larvae were blown into the water and fed with paramecium for constant temperature culture at 28α. The survival curves of the larvae were monitored once a day at regular intervals. Juvenile fish that were physically damaged by microinjection and died within 48 h post-infection were excluded.

Six-month-old zebrafish (*AB/TU*) were obtained from Company EzeRinka and were infected by control and knock-down strains, respectively (1,000 CFU; 35 per group) by intraperitoneal injection. Five fish per group were anesthetized with tricaine (500 mg/L) at fortnight post-infection, fixed in 4% formalin, and then dehydrated with different concentrations of ethanol. After paraffin embedding, sections were stained with H&E staining and Ziehl-Neelsen acid fast staining. Concurrently, additional zebrafish were sacrificed ethically and dispersed in homogenate tubes. The samples were diluted with PBS on the plate (kanamycin 25 μg/ml) to count the CFU.

### Murine tail infection and histology

The mammalian body temperature is too high for the growth of *M. marinum*, so the murine tail infection model with a low temperature is an ideal infection model (77, 78). Female C57BL/6 mice (five mice per group) were inoculated with 3 × 10^7^ CFUs in the tail vein. Ulceration of the tails was observed every day post-infection. After a week, the visible lesion parts were cut away and embedded into sections with the bones removed. The pathological results and the inflammatory cell infiltration were observed by H&E staining following standard protocols. The specimens were subjected for analysis by an independent pathologist who was blinded to the infection results to reduce bias. The presence of inflammatory cells (macrophages, plasma cells/lymphocytes, neutrophils, and eosinophils) in addition to the degree of inflammation (granulomas, calcification, and vasculitis), tissue damage (dermal and fat tissue necrosis and muscle layer involvement), and vascular involvement was scored. Specimens from two noninfected mice were used as controls.

The animal experiment procedure was reviewed and approved by the Animal Care and Use Committee of China Three Gorges University.

### Transcriptome profiling

Murine macrophages (cell line J774A.1) infected by the control and knock-down strains (MOI = 5) were collected 12 h post-infection by TRIzol (Invitrogen), and the transcriptomes were purified. RNAseq was performed by Illimina (Majorbio bioinformatic platform), and a total of 71.78 GB of cleaned data was obtained. The reference gene source was Mus_musculus (GRCM38.p6, http://asia.ensembl.org/Mus_musculus/Info/Index). Clean reads of each sample were sequenced, and the alignment rates ranged from 95.25 to 95.7%. Functional database and annotation analysis (NR, Swiss-Prot, PFAM, COG, GO, and KEGG) were conducted for the expressed genes and transcripts. The DEG analysis software was edgeR, and the screening threshold was |log_2_FC| ≥1 and *p*-value <0.05. The software Goatools was used for GO enrichment analysis by Fisher’s exact test, and KEGG pathway enrichment analysis was performed by R-scripts. When a corrected *P*-value (*P*-adjust) was <0.05, the enrichment was considered significant. Matching RNA samples were used for quantitative PCR validation, and the primers are given in **Supplementary Table S3**.

### Cytokine analysis

Culture supernatants of murine macrophages (J774A.1 cell line) infected by the control and knock-down strains (MOI = 5) were collected at 12 h post-infection. Liquid suspension protein microarray (Bio-PlexTM 200 System, Bio-Rad) and the kit Bio-plex ProTM Mouse Cytokine Grp 1 Panel 23-plex (Bio-Rad) were used to detect the cytokine secretions in supernatants.

## Data availability statement

The original contributions presented in the study are publicly available. This data can be found here: https://www.ncbi.nlm.nih.gov/search/all/?term=GSE197488

## Ethics statement

This study was reviewed and approved by the Animal Care and Use Committee of China Three Gorges University.

## Author contributions

Design of research studies: LZ, H-PL, JL, and Y-YL. Experiments and data acquisition: Y-YL, H-ML, L-SZ, CD, Z-ZW, CL, X-YW, L-HQ, S-QX, DW, and YL. Data analysis: LZ, Y-YL, DW, YLu, and YLi. Original idea and writing of the manuscript: Y-YL, H-PL, JL, and LZ. Manuscript revision: LZ, Y-YL, H-PL, and JL. All authors contributed to the article and approved the submitted version.

## Funding

This study was supported by grants from the National Key Research and Development Program of China (2021YFC2301500), the National Key Research and Development Program of China (no. 2018YFD0500900) and the National Natural Science Foundation of China (no. 31772709).

## Conflict of interest

The authors declare that the research was conducted in the absence of any commercial or financial relationships that could be construed as a potential conflict of interest.

## Publisher’s note

All claims expressed in this article are solely those of the authors and do not necessarily represent those of their affiliated organizations, or those of the publisher, the editors and the reviewers. Any product that may be evaluated in this article, or claim that may be made by its manufacturer, is not guaranteed or endorsed by the publisher.

## Supplementary material

Supplementary Figure S1 Vector map pf CRISPRi backbone-PLJR962. ATc as the small molecule regulator inactivates the Tet repressor and induces the expression of dCas9, which is directed to specific DNA targets by ATc-inducible sgRNA. sgRNA-coding sequence is inserted into the Sthe sgRNA scaffold position. The SapI-based Golden Gate handle allows for the cloning of additional eight sgRNA cassettes into the backbone. Double gene knock-down (GlfT1+GlfT2) strain fail to be constructed because of off-target effect which is unavoidable in CRISPRi.

Supplementary Figure S2
**(A)** Relative expressions of corresponding genes in knock-down strains (AftC/EmbB/GlfT1_KD) were validated by qPCR. All single and combinational double knock-down vectors were constucted, only the strains with significant knock-down were shown due to the off-target effects of sgRNA, with a 20% to 30% reduction in relative gene expression. Data are plotted as mean ± SD, unpaired t test, EmbB_KD (*: p value =0.0278); GlfT1_KD (*:p value=0.0305), AftC_KD (**: p value=0.0064). **(B)** AG biosynthesis genes relative expressions of the control strain verified by qPCR. Data are plotted as mean ± SD. Two-way ANOVA tests are used for all significance analysis and no significant change in genes expressions under ATc induction. **(C)** Growth curves of EmbA/GlfT2_KD strains with or without ATc induction.**(D)** Relative genes expressions of EmbA_KD strains qualified by qPCR. Data are plotted as mean ± SD. Two-way ANOVA tests are used for all significance analysis, ***: p value =0.0001; ****: p value <0.0001. **(E)** Relative genes expressions of GlfT2_KD strains qualified by qPCR. Data are plotted as mean ± SD. Two-way ANOVA tests are used for all significance analysis, ***: p value =0.0001.

Supplementary Figure S3 The content of cytokines in the cell supernatant of macrophage J774A.1 infected by control PLJR962 strain and EmbA_KD stains with MOI=5. Two-way ANOVA tests are used for all significance analysis here; **: P <0.01; **: P <0.01; ***: P <0.001; ****: P <0.0001.

Supplementary Figure S4
**(A)** Uptake of knock-down strains by human THP-1 cells and shGalectin9 (Galcetin9 knock-out on the basis of THP-1) cells when cells infected with MOI=5 48h post infection. **(B, C)** Intracellular CFU counts of EmbA_KD strain when shGalectin9 cells infected with MOI=5 48h post infection. Data are plotted as mean ± SD. Two-way ANOVA tests are used for all significance analysis here; **: P <0.01; **: P <0.01; ***: P <0.001; ****: P <0.0001.
