# Supplementary figures and images for: Arabinogalactan enhances Mycobacterium marinum virulence by suppressing host innate immune responses
Source: Front Immunol. 2022 Aug 26;13:879775. doi: 10.3389/fimmu.2022.879775 (PMC9459032; doi:10.3389/fimmu.2022.879775)

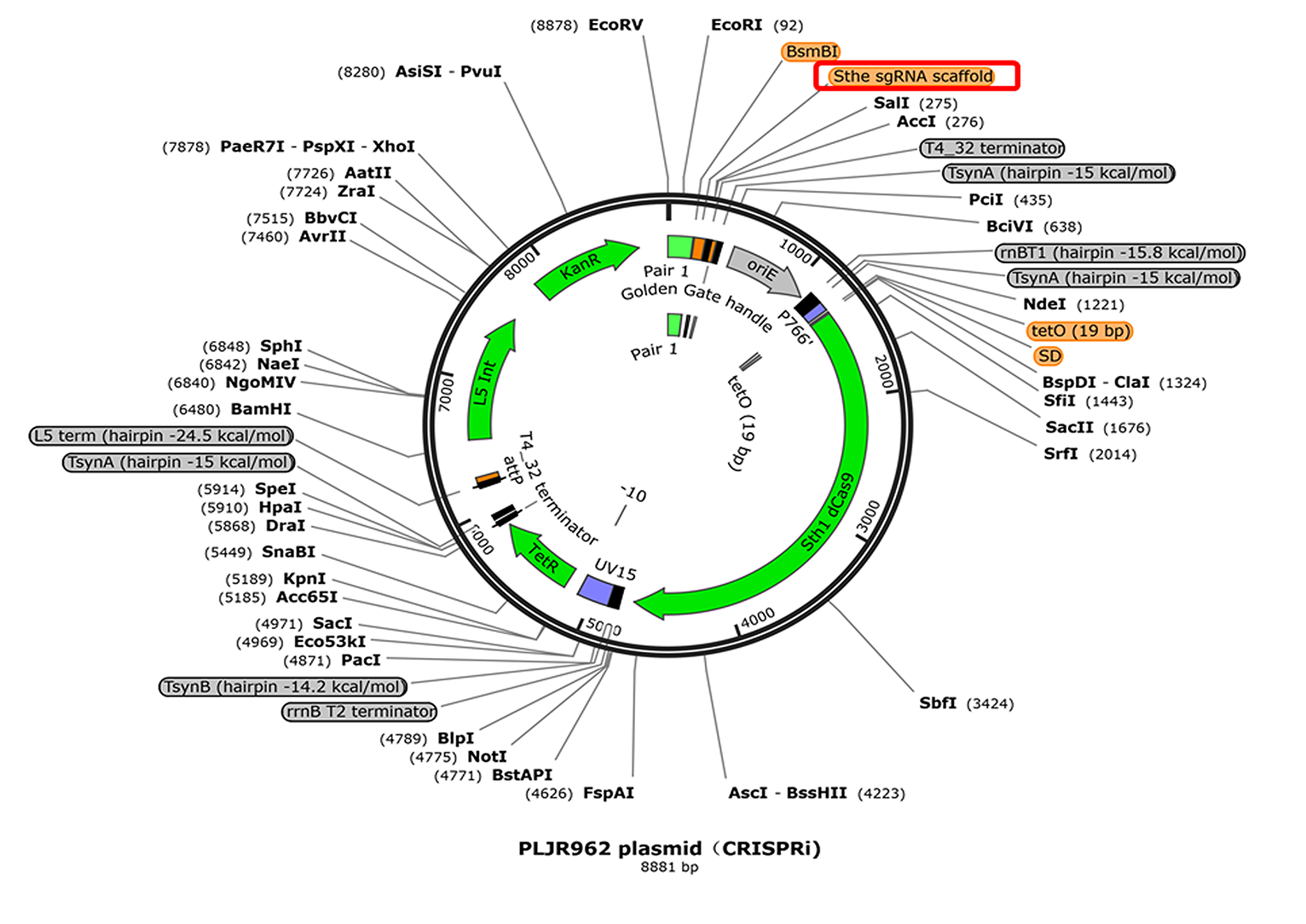

Supplement: Supplementary Figure S1 — Vector map pf CRISPRi backbone-PLJR962. ATc as the small molecule regulator inactivates the Tet repressor and induces the expression of dCas9, which is directed to specific DNA targets by ATc-inducible sgRNA. sgRNA-coding sequence is inserted into the Sthe sgRNA scaffold position. The SapI-based Golden Gate handle allows for the cloning of additional eight sgRNA cassettes into the backbone. Double gene knock-down (GlfT1+GlfT2) strain fail to be constructed because of off-target effect which is unavoidable in CRISPRi. [file Image_1.tif]

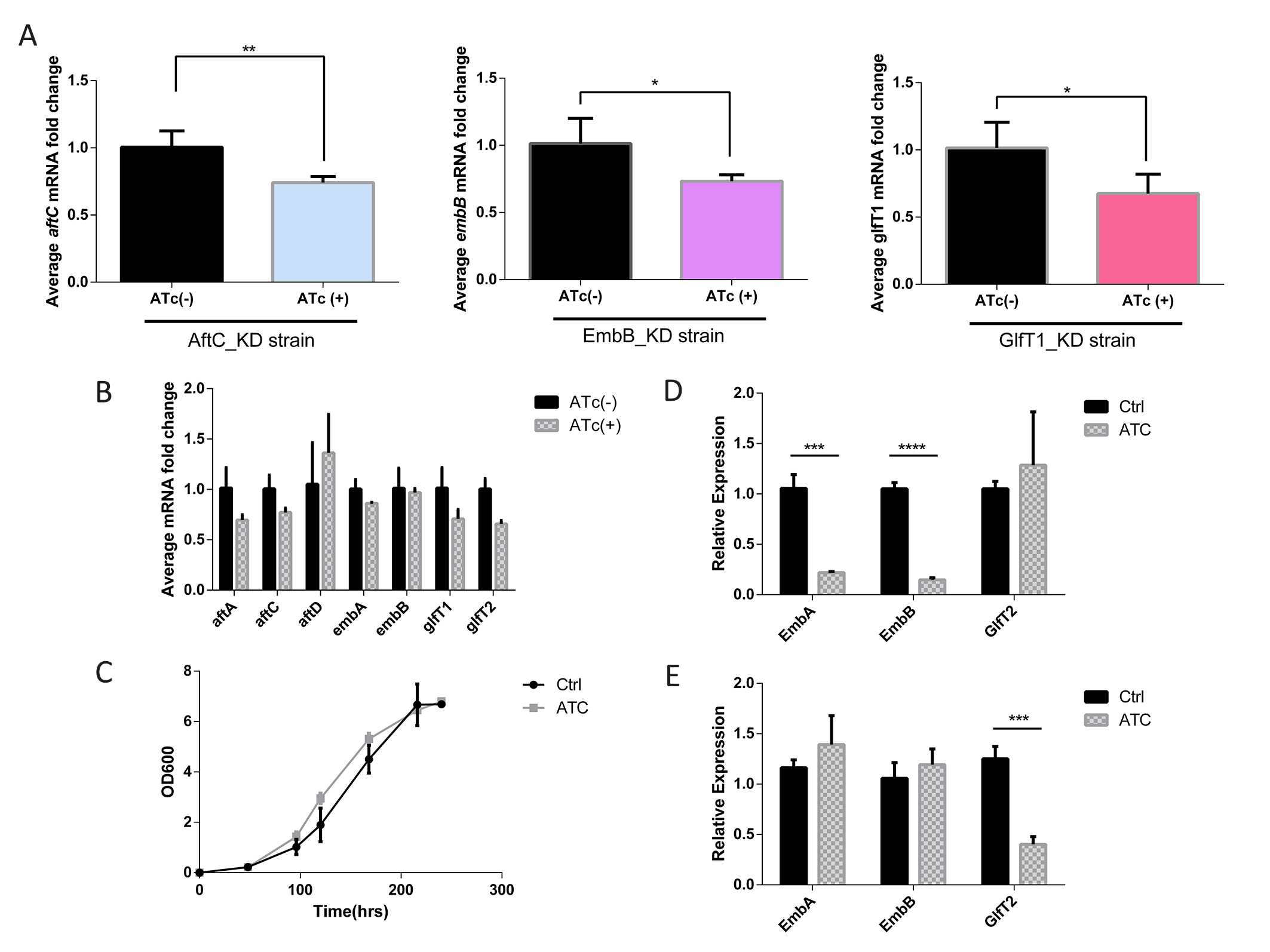

Supplement: Supplementary Figure S2 — (A) Relative expressions of corresponding genes in knock-down strains (AftC/EmbB/GlfT1_KD) were validated by qPCR. All single and combinational double knock-down vectors were constucted, only the strains with significant knock-down were shown due to the off-target effects of sgRNA, with a 20% to 30% reduction in relative gene expression. Data are plotted as mean ± SD, unpaired t test, EmbB_KD (*: p value =0.0278); GlfT1_KD (*:p value=0.0305), AftC_KD (**: p value=0.0064). (B) AG biosynthesis genes relative expressions of the control strain verified by qPCR. Data are plotted as mean ± SD. Two-way ANOVA tests are used for all significance analysis and no significant change in genes expressions under ATc induction. (C) Growth curves of EmbA/GlfT2_KD strains with or without ATc induction.(D) Relative genes expressions of EmbA_KD strains qualified by qPCR. Data are plotted as mean ± SD. Two-way ANOVA tests are used for all significance analysis, ***: p value =0.0001; ****: p value <0.0001. (E) Relative genes expressions of GlfT2_KD strains qualified by qPCR. Data are plotted as mean ± SD. Two-way ANOVA tests are used for all significance analysis, ***: p value =0.0001. [file Image_2.tif]

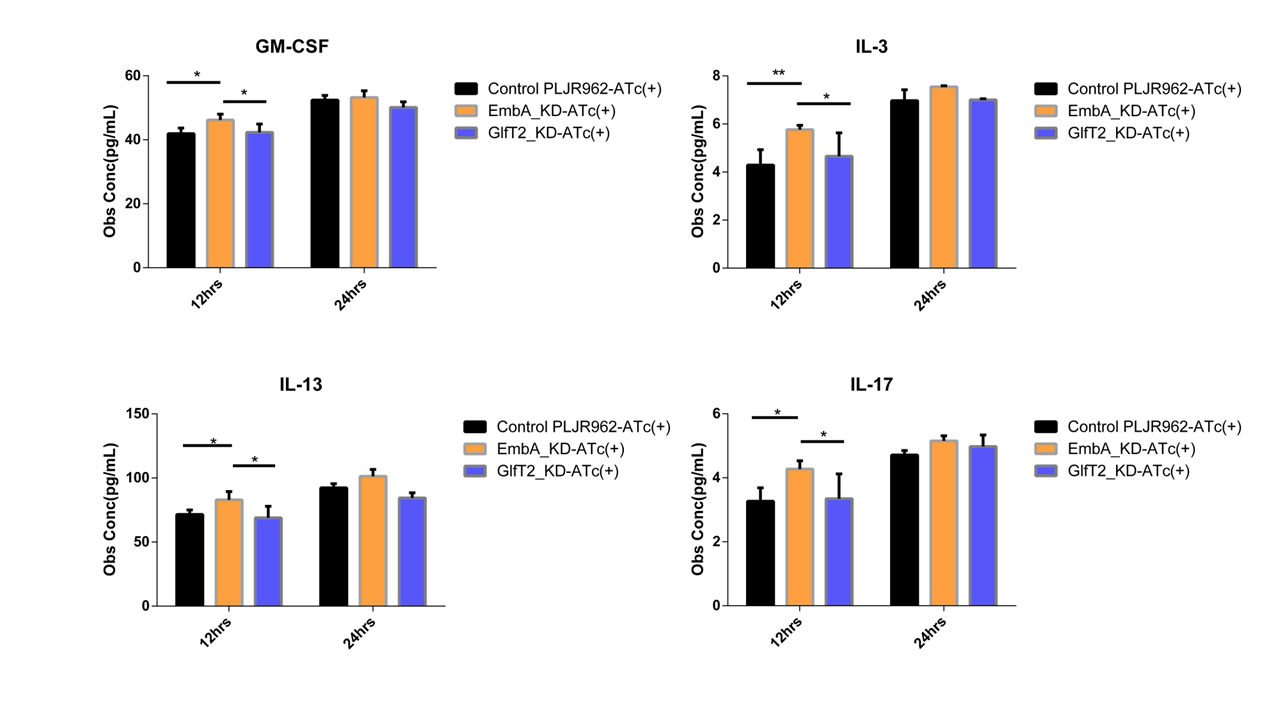

Supplement: Supplementary Figure S3 — The content of cytokines in the cell supernatant of macrophage J774A.1 infected by control PLJR962 strain and EmbA_KD stains with MOI=5. Two-way ANOVA tests are used for all significance analysis here; **: P <0.01; **: P <0.01; ***: P <0.001; ****: P <0.0001. [file Image_3.tif]

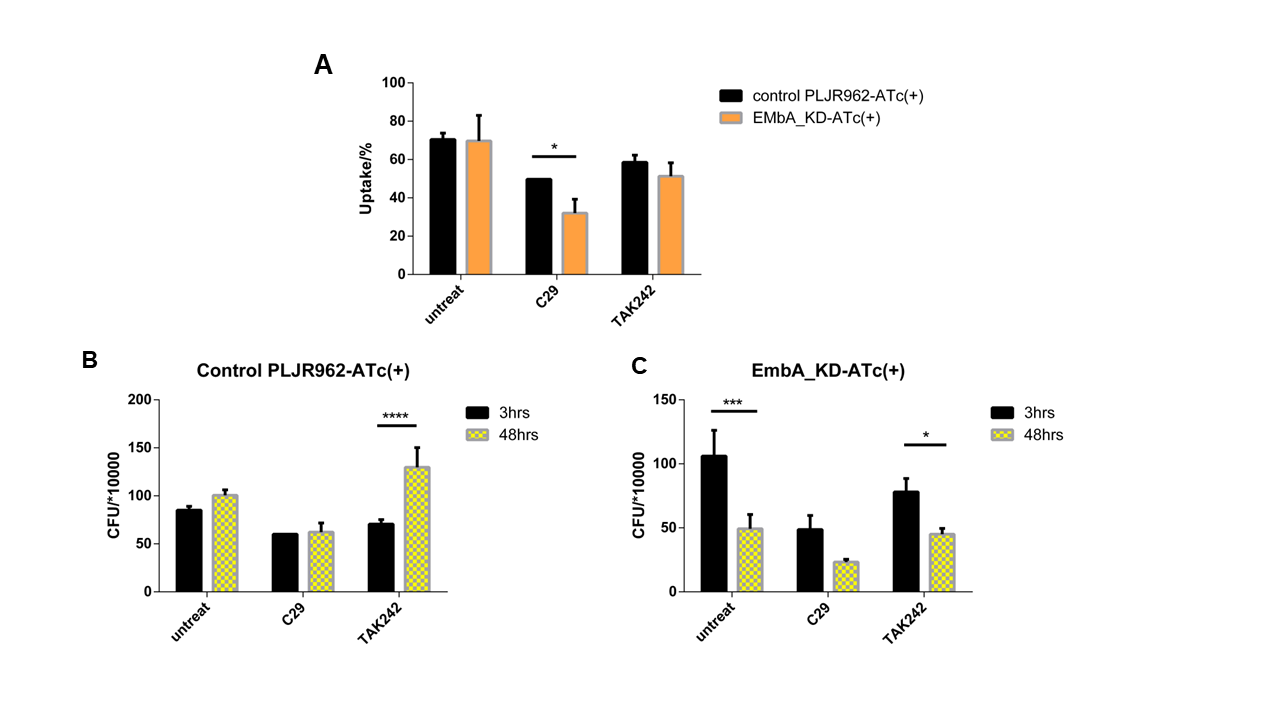

Supplement: Supplementary Figure S4 — (A) Uptake of knock-down strains by human THP-1 cells and shGalectin9 (Galcetin9 knock-out on the basis of THP-1) cells when cells infected with MOI=5 48h post infection. (B, C) Intracellular CFU counts of EmbA_KD strain when shGalectin9 cells infected with MOI=5 48h post infection. Data are plotted as mean ± SD. Two-way ANOVA tests are used for all significance analysis here; **: P <0.01; **: P <0.01; ***: P <0.001; ****: P <0.0001. [file Image_4.tif]
