# Supplementary material for: Arabinogalactan enhances Mycobacterium marinum virulence by suppressing host innate immune responses
Source: Front Immunol. 2022 Aug 26;13:879775. doi: 10.3389/fimmu.2022.879775 (PMC9459032; doi:10.3389/fimmu.2022.879775)
Supplement: Supplementary file 5 [file Table_1.docx]

**Supplementary Table 1 Primers contained sgRNA-coding sequence for construction of CRISPRi backbones.**

| Gene name | Locus（M.marium） | Primer Sequence |
| --- | --- | --- |
| *glfT1* | *MMAR_5337* | F:5’-GGGAGcgctgttccgcgcgtcgacg-3’  R:5’-AAACcgtcgacgcgcggaacagcgC-3’ |
| *glfT2* | *MMAR_5372* | F:5’-GGGAGcagggtcatgtacgaggcgct-3’  R:5’-AAACagcgcctcgtacatgaccctgC-3’ |
| *embA* | *MMAR_5356* | F:5’-GGGAGcgccgggggcgtggataccgg-3’  R:5’-AAACccggtatccacgcccccggcgC-3’ |
| *embB* | *MMAR_5357* | F:5’-GGGAGtgatgacccacgtcagcgacg-3’  R:5’-AAACcgtcgctgacgtgggtcatcaC-3’ |
| *aftC* | *MMAR_2043* | F:5’-GGGAGcgcggctgggacatctataa-3’  R:5’-AAACttatagatgtcccagccgcgC-3’ |
