# Supplementary material for: Arabinogalactan enhances Mycobacterium marinum virulence by suppressing host innate immune responses
Source: Front Immunol. 2022 Aug 26;13:879775. doi: 10.3389/fimmu.2022.879775 (PMC9459032; doi:10.3389/fimmu.2022.879775)
Supplement: Supplementary file 6 [file Table_2.docx]

**Supplementary Table S2- MIC (minimum inhibitory concentrations) assay**

Resistance of control PLJR962 strain and EmbA/GlfT2_KD strains with or without ATc induction.

|  | **Ethambutol**  **(μg/mL)** | **Isoniazid（μg/mL）** |
| --- | --- | --- |
| **Control-PLJR962-ATc(-)** | **20** | **200** |
| **Control-PLJR962-ATc(+)** | **20** | **200** |
| **EmbA_KD-ATc(-)** | **20** | **200** |
| **EmbA_KD-ATc(+)** | **5.24** | **200** |
| **GlfT2_KD-ATc(-)** | **20** | **200** |
| **GlfT2_KD –ATc(+)** | **20** | **200** |
