# Supplementary material for: Arabinogalactan enhances Mycobacterium marinum virulence by suppressing host innate immune responses
Source: Front Immunol. 2022 Aug 26;13:879775. doi: 10.3389/fimmu.2022.879775 (PMC9459032; doi:10.3389/fimmu.2022.879775)
Supplement: Supplementary file 7 [file Table_3.docx]

**Supplementary Table S3 Primers used for quantitative real time PCR analysis**

| Gene name | Forward primer | Reverse primer |
| --- | --- | --- |
| *sigA* | 5’-CGACGATCTCGACCTCGACTTGGATGAC-3’ | 5’-GCTGGGCTCGGCGATCTCTTCTTCA-3’ |
| *glfT1* | 5’-GGGTCCGACGAGTTCAAACCGATCCT-3’ | 5’-AAGAACCAGCCGAACCTCAGCCACT-3’ |
| *glfT2* | 5’-GTGTATCGGGCGGTGCTCAACTTCC-3’ | 5’-GCGGTGTTGATCGAGATGAACAGGTAGC-3’ |
| *embA* | 5’-AGGCGGCACGGATCAAGTACAAGG-3’ | 5’-AGCACCACCAGTGAGCCGAACA-3’ |
| *embB* | 5’-GCTCGTTGCAGGAGTACGTCGGTTC-3’ | 5’-CGGTGTCCAGCTTCTTGGCGTTGTAG-3’ |
| *aftA* | 5’-CGGCACTGCACGACATGACCTACA-3’ | 5’-GAATCATTCGCCACCACAGCACCAG-3’ |
| *aftC* | 5’-GTGTATCGGGCGGTGCTCAACTTCC-3’ | 5’-GCGGTGTTGATCGAGATGAACAGGTAGC-3’ |
| *aftD* | 5’-CACCAAGCTCGATCTCACCGCCAATC-3’ | 5’-GCAGCAGGTGTCCGACCAGGAAGAA-3’ |
| *gapdh* | 5’-GTGAAGGTCGGTGTGAACGGATT-3’ | 5’-GGTCTCGCTCCTGGAAGATGGT-3’ |
| *cebpb* | 5’-TGGACAAGCTGAGCGACGAGTA-3’ | 5’-AGCTGCTCCACCTTCTTCTGC-3’ |
